# Comparison of Biological and Mechanical Prostheses for Heart Valve Surgery: A Systematic Review of Randomized Controlled Trials

**DOI:** 10.5935/abc.20180272

**Published:** 2019-03

**Authors:** Alberto Takeshi Kiyose, Erica Aranha Suzumura, Lígia Laranjeira, Anna Maria Buehler, José Amalth Espírito Santo, Otavio Berwanger, Antonio Carlos de Camargo Carvalho, Angelo Amato de Paola, Valdir Ambrósio Moises, Alexandre Biasi Cavalcanti

**Affiliations:** 1 Universidade Federal de São Paulo (UNIFESP), São Paulo, SP - Brazil; 2 Hospital do Coração (HCOR), São Paulo, SP - Brazil

**Keywords:** Heart Valve Prosthesis, Bioprosthesis, Metal-on-Metal Joint Prosthesis, Heart Valve Prosthesis Implantation/trends, Review

## Abstract

**Background:**

The choice of a mechanical (MP) or biological prosthesis (BP) for patients with valvular heart disease undergoing replacement is still not a consensus.

**Objective:**

We aimed to determine the clinical outcomes of MP or BP placement in those patients.

**Methods:**

We conducted a systematic review and meta-analysis of randomized controlled trials (RCTs) that compared biological prostheses and mechanical prostheses in patients with valvular heart diseases and assessed the outcomes. RCTs were searched in the MEDLINE, EMBASE, LILACS, CENTRAL, SCOPUS and Web of Science (from inception to November 2014) databases. Meta-analyses were performed using inverse variance with random effects models. The GRADE system was used to rate the quality of the evidence. A P-value lower than 0.05 was considered significant.

**Results:**

A total of four RCTs were included in the meta-analyses (1,528 patients) with follow up ranging from 2 to 20 years. Three used old generation mechanical and biological prostheses, and one used contemporary prostheses. No significant difference in mortality was found between BP and MP patients (risk ratio (RR = 1.07; 95% CI 0.99-1.15). The risk of bleeding was significantly lower in BP patients than MP patients (RR = 0.64; 95% CI 0.52-0.78); however, reoperations were significantly more frequent in BP patients (RR = 3.60; 95% CI 2.44-5.32). There were no statistically significant differences between BP and MP patients with respect to systemic arterial embolisms and infective endocarditis (RR = 0.93; 95% CI 0.66-1.31, RR = 1.21; CI95% 0.78-1.88, respectively). Results in the trials with modern and old prostheses were similar.

**Conclusions:**

The mortality rate and the risk of thromboembolic events and endocarditis were similar between BP and MP patients. The risk of bleeding was approximately one third lower for BP patients than for MP patients, while the risk of reoperations was more than three times higher for BP patients.

## Introduction

In the early 1960's, valve replacement surgery using prostheses completely changed the natural history of patients with valvular heart disease. Approximately 90,000 valve prostheses are implanted in the United States, and 280,000 are implanted worldwide each year.^1^ Currently, the total number of biological valve prosthesis implants surpasses that of mechanical prosthesis implants.^2^^-^^4^

The factors that seem to affect the increased use of biological prostheses include advances in their construction, leading to increased durability, and the fact that they do not require permanent use of oral anticoagulants.^5^ However, biological prostheses still present an increased risk of structural deterioration and the need for reoperation, although the surgical risk involved in reoperation has decreased substantially in recent years.^6^ Furthermore, in the event of a stenosis disorder, patients with aortic bioprosthesis impairment can be treated with a catheter-implanted prosthesis.^7^

A systematic review of randomized trials published in 2000 comparing mechanical and biological valve prostheses suggested that no difference in mortality existed between the two implant types.^8^ There was, however, less risk of reoperation with mechanical prostheses but increased risk of bleeding compared to biological prostheses. There are no recent systematic reviews comparing the performance of biological valve prostheses with that of mechanical prostheses. Since the publication of the last review, further randomized studies may have been published that better reflect progress in prosthesis development, surgical techniques and clinical treatments during that time period. The objective of the present systematic review of randomized studies was to compare the effect of biological valve prosthesis use with that of mechanical prosthesis use in terms of mortality, reoperations, the incidence of thromboembolic events, bleeding, and endocarditis.

## Methods

### Search strategy and sources

The literature search included the following electronic databases: MEDLINE/PubMed, (from 1950 to 04 November 2014), CENTRAL/Cochrane Library, EMBASE/Elsevier (from 1966 to November 4, 2014), SCOPUS/Elsevier (from 1960 to November 4 2014), Web of Science/Thomson Reuters (from 1898 to November 4, 2014), and LILACS/BVS (from 1980 to November 4, 2014), without language and publication date restrictions. Previous systematic reviews and guidelines were consulted to identify and include relevant studies. Other sources were also consulted to identify relevant studies including Clinicaltrials.gov, conference abstracts; lists of text references related to the topic; review articles; and information letters concerning unpublished or incomplete studies.

The search strategies were developed by defining descriptors, synonyms and the use of Boolean logical operators (AND, OR, and AND NOT) for each database (MeSH/Medline, Emtree/Embase, and DeCs/BVS).^9^ The MeSH/Medline subject descriptors were sensitised by the strategy of adding "entry terms" (synonyms). In Medline, the Cochrane Handbook Filter^10^ was used, which has high sensitivity for recovery of indexed randomized controlled trials (RCTs).

### Study Selection

We included randomized trials in any language that compared native valve replacement with the biological and mechanical prosthesis, regardless of the follow-up period. Observational studies, studies with children or patients under 18 years of age, and studies with patients who required tricuspid valve replacement were excluded. The study eligibility evaluation process consisted of two steps, both performed independently by pairs of reviewers. The first author (ATK) participated in all pairs. The first step consisted of screening articles by reading the title and abstract. In this step, the article was selected for the next step if at least one of the reviewers deemed the article eligible. In the second step, the full article texts were evaluated and selected based on an eligibility form. The final eligibility of the article was decided by agreement between the reviewers or by the judgment of a third reviewer in the event of a disagreement. In the case of multiple publications of the same study, we considered the manuscript reporting the longest follow-up.

### Data extraction and risk of bias

For the data extraction process, we developed a standard form with the clinical information of each patient, including gender, age, functional class, affected valve, type of implanted prosthesis, follow-up period, and methodological characteristics, for further evaluation of evidence quality.

An assessment of the risk of bias of the included studies was based on an evaluation of the following domains: random sequence generation, allocation concealment, blinding of outcome assessors, and incomplete outcome data. Blinding of patients and the healthcare team regarding the prosthesis type was not feasible, and these items were therefore not evaluated. We generated a descriptive table to compare the selected studies by classifying the risk of bias as low, moderate, high, or unclear for each risk of bias domain.

### Outcomes

The outcomes measured included total mortality, defined as death from any cause; embolic events, defined as a systemic embolism; bleeding events (of any magnitude); new surgery, defined as the need to replace the prosthesis implanted in the initial procedure; and episodes of infectious endocarditis.

### Data synthesis and analysis

We determined the risk ratios (RRs) and their respective 95% confidence intervals (CIs) for binary outcomes of each trial. Meta-analyses were performed with random effects models using inverse variance. Subgroup analyses were conducted based on the position of valve replacement (aortic, mitral or combined aortic-mitral).

Most trials did not report the number of events, only probabilities of events and their standard errors. Thus we calculated the variance of the logarithm of the RR with the formula used by Kassai et al.^8^

$$\frac{{\mathrm{SE}}_{1}^{2}}{P_{1}^{2}}+\frac{{\mathrm{SE}}_{2}^{2}}{P_{2}^{2}}$$

Where:

p_1_ = the probability of an event for a mechanical heart valve

p_2_ = the probability of an event for a bioprosthesis

SE_1_ = standard error of p_1_

SE_2_ = standard error of p_2_

We assessed the statistical heterogeneity across trials or subgroups using Cochrane's chi-squared test. The Higgins inconsistency test (I^2^) was used to quantify the percentage of the variability in the effect estimates that was due to heterogeneity rather than by chance;^11^ we considered values of I^2^ ≤ 25% as low heterogeneity and values ≥ 50% as high heterogeneity. We conducted these analyses using Review Manager Version 5.2 software (Cochrane IMS, Oxford, UK). A p-value lower than 0.05 was considered significant.

### Quality of evidence assessment

We assessed the confidence in the estimates of effect (quality of evidence) using the GRADE (Grades of Recommendation, Assessment, Development, and Evaluation) system.^12^

## Results

### Characteristics of included studies

**Figure 1:**
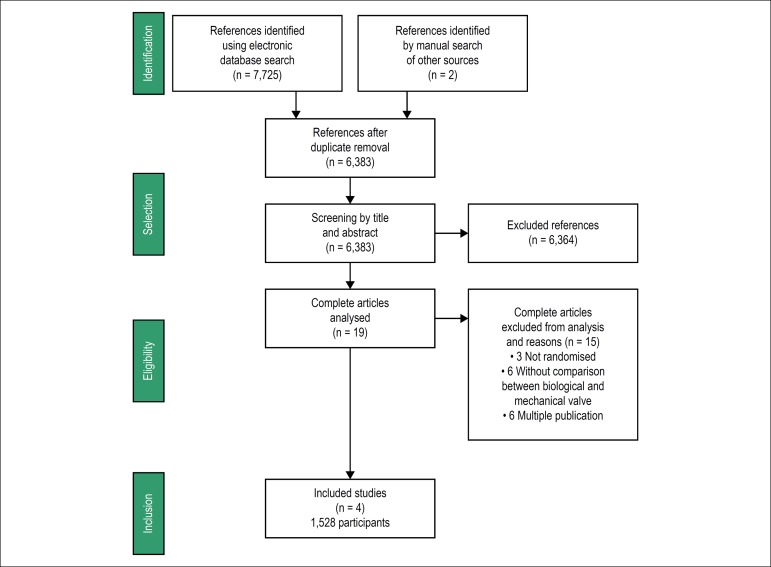
Study search and selection processes.

The electronic database search resulted in 7,725 citations (Figure 1). After evaluation of the articles, we identified four original studies including 1,528 patients in total. The clinical characteristics of the four included studies are presented in Table 1.

**Table 1:** Characteristics of included studies

Trials	Year of publication	Total randomised	Type of valves	Number Randomised	Patients Characteristics	Local of prosthesis implantation	Follow-up (m/y)
Vallejo	1981	110	Bioprosthesis: Angell-Shiley	38	7% NYHA II; 27% NYHA III; 4% NYHA IV 66% Male, Mean age: 39.7 ± 11 .2	MVR	Mean 24.13 ±11.16 m
			Mechanical prosthesis *: Bjork-Shiley	35	7% NYHA II; 24% NYHA III; 4% NYHA IV 69% Male, 40.7 ± 11.3	MVR	Mean 31.61 ± 13.02 m
			Lillehei-Kaster	37	4% NYHA II; 30% NYHA III; 3% NYHA IV 76% Male, 41.9 ± 10.4	MVR	Mean 30.4 ± 15.9 m
Veterans Affairs (Hammermeister)	2000	575	Bioprosthesis: Hancock porcine	289	100% Male	67% AVR; 33% MVR	Maximum 18 y
			Mechanical prosthesis: Bjork-Shiley	286	100% Male	69% AVR; 31% MVR	Maximum 18 y
Edinburgh (Oxenham, Bloomfield)	2003	533	Bioprosthesis: Hancock porcine Carpentier- Edwards	107 159	53% NYHA III or IV AF † 76% Female mitral valve	38% AVR, 50% MVR, 12% AVR+MVR	Mean 20.4 y
			Mechanical prosthesis: Bjork-Shiley	267	57% NYHA III or IV 74% Female mitral valve	41% AVR, 48% MVR, 11% AVR+MVR	Mean 20.4 y
Stassano	2009	310	Bioprosthesis: Carpentier- Edwards SAV	93	75.5% NYHA III or IV Male 50.3% Age 63.5 ± 3.9	100% AVR	Mean 106 ± 28 m
			Carpentier- Edwards Pericardial	62			
			Mechanical prosthesis: St. Jude Medical	107	76,8% NYHA III or IV Male 42,5% Age 64.0 ± 7.6		
			Carbomedics	48			

* Tilting disc valve. 37.8% previous surgery in mitral valve with LK (p < 0.005);

† 67% Bioprosthesis in atrial fibrillation.

Vallejo et al.^13^ randomized 110 mitral valve replacement candidates, from 1975 to 1979, into one of three groups: Angell-Shiley porcine bioprosthesis, Björk-Shiley mechanical prosthesis, and Lillehei-Kaster mechanical prosthesis. The mean follow-up time was approximately two years.^13^

The Veterans Affairs Cooperative Study randomized 575 patients between 1977 and 1982.^14^^-^^17^ This study included men who received a Hancock first generation porcine bioprosthesis or Björk-Shiley mechanical single spherical 60 degrees disc prosthesis. Most patients (70%) underwent aortic valve replacement. The mean follow-up time was 15 years.

Bloomfield et al randomized 533 patients of both genders to receive either a mechanical Byork-Schiley 60 degrees spherical tilting disk or a porcine bioprosthetic valve. Between 1975 and 1977 the patients assigned to a bioprosthesis received a Hancock prosthesis and after January 1977 to 1979 such patients received a Carpentier- Edwards prosthesis.^18^^-^^20^ Approximately half of the patients underwent aortic valve replacement, and half underwent mitral valve replacement. The mean follow-up time was 20 years.

Stassano et al.^21^ randomized 310 patients, who required aortic valve replacement between 1995 and 2003, into a biological prosthesis group and a mechanical prosthesis group.^21^ Carpentier-Edwards porcine or Carpentier-Edwards bovine pericardial prostheses were used in the bioprosthesis group. In the group allocated to mechanical prostheses, Carbomedics or St. Jude double disc prostheses were used. The mean follow-up time was 8.8 years.

### Risk of Bias

Characteristics related to the risk of bias of the studies are presented in Table 2. None of the studies described how the random list was generated. The trials were at low risk of bias for all the other domains including allocation concealment, blinding of outcome assessors, and incomplete outcome data. None of the studies used blinding of patients and health professionals, which is not feasible in this scenario.

**Table 2:** Risk of bias in included studies

	Vallejo 1981	Veterans 2000	Edinburgh 2003	Stassano 2009
Random sequence generation	Unclear	Unclear	Unclear	Unclear
Allocation concealment	Low risk of bias	Low risk of bias	Low risk of bias	Low risk of bias
Blinding of outcome assessors	Low risk of bias	Low risk of bias	Low risk of bias	Low risk of bias
Complete outcome data	Low risk of bias	Low risk of bias	Low risk of bias	Low risk of bias

### Clinical outcomes

**Figure 2:**
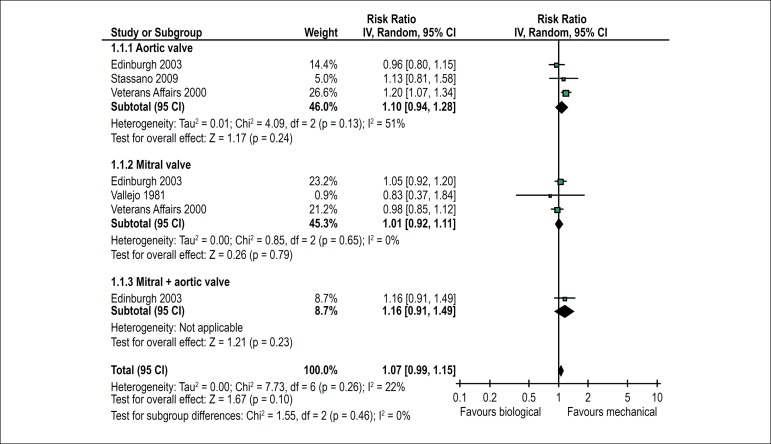
Forest plot showing the effects of biological versus mechanical prostheses on mortality.

There was no statistically significant difference in the risk of death between biological or mechanical prosthesis, although most of the confidence interval favours the latter (RR = 1.07; 95% CI 0.99-1.15) (Figure 2). In addition, mortality was similar in the subgroups of patients receiving prostheses in the aortic or mitral positions or in both positions simultaneously. The effect estimates from different studies were reasonably homogeneous (I² = 22%).

**Figure 3:**
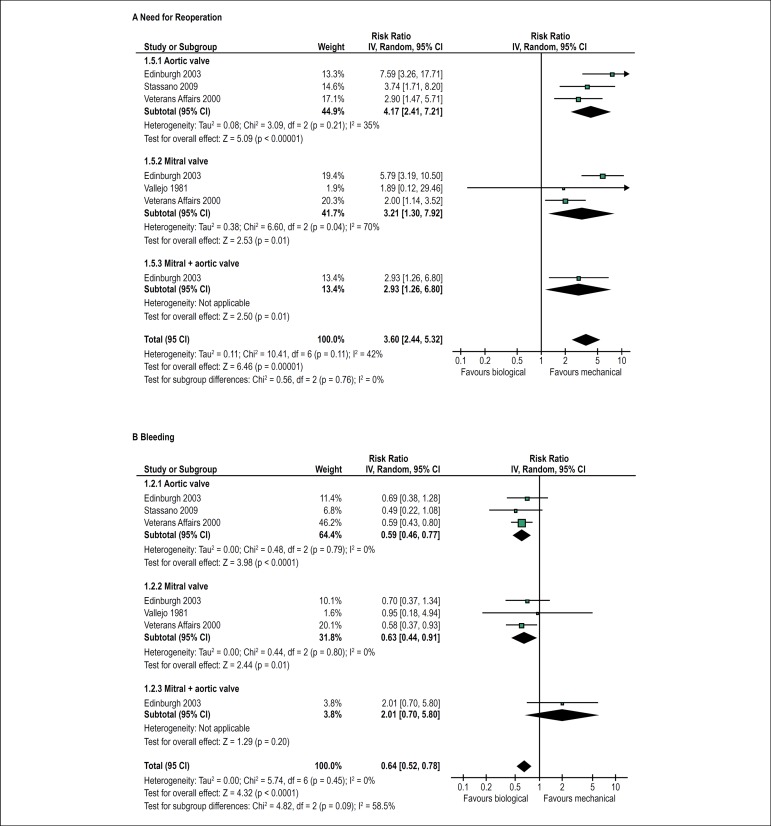
Forest plots showing the effects of biological versus mechanical prostheses on a need for reoperation (A) and risk of bleeding (B).

The need for reoperation was more frequent among patients who received biological prostheses than among those who received mechanical prostheses (RR = 3.60; 95% CI 2.44-5.32; I^2^ = 0%). The effect was similar in patients who received prostheses in the aortic or mitral position or both simultaneously (Figure 3).

The risk of bleeding was lower in patients treated with biological prostheses than in those treated with mechanical prostheses (RR = 0.64; 95% CI 0.52-0.78; I^2^ = 0%). There was a trend toward a distinct effect between the subgroups according to the position of the implant, but that was not statistically significant (P for subgroup differences = 0.09) (Figure 3). It should be noted that the definitions of bleeding were not equal across studies. Vallejo et al.^13^ considered only bleeding that required hospitalisation or that was a direct cause of death.^13^ In their study, Bloomfield et al.^20^ included all major (65%) and minor bleeding.^20^ The Veterans Affairs study included clinically important bleeding.^17^ Stassano et al.^21^ made no reference to the magnitude of the bleeding.^21^

**Figure 4:**
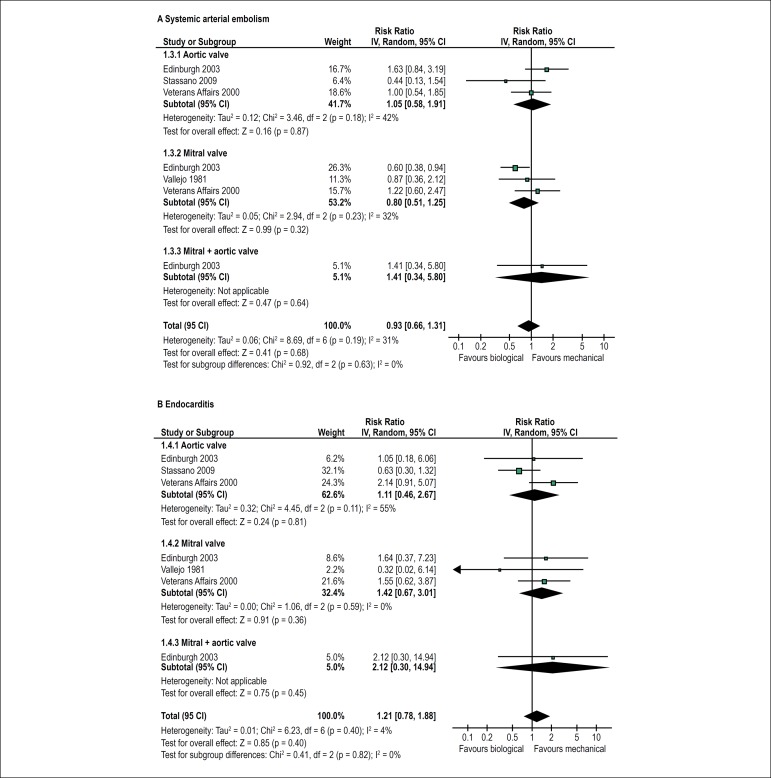
Forest plots showing the effects of biological versus mechanical prostheses on the risk of systemic arterial embolism (A) and the risk of endocarditis (B).

There were no significant differences in the risk of endocarditis (RR = 1.21, 95% CI 0.78-1.88; I^2^ = 4%) or systemic arterial embolism (RR = 0.93, 95% CI 0.66-1.31; I^2^ = 31%) between the group that received bioprostheses and the group that received mechanical prostheses (Figure 4).

## Discussion

This systematic review and meta-analysis of randomized studies involving patients requiring cardiac valve replacement revealed similar mortality between patients who underwent implantation of biological prostheses and those who underwent implantation of mechanical prostheses. There were also no differences regarding the risk of thromboembolism and endocarditis. However, the risk of bleeding was approximately one third lower among patients treated with biological prostheses than in those treated with mechanical prostheses. In contrast, the need for reoperation among patients treated with bioprostheses was more than three times greater that of patients treated with mechanical prostheses.

Currently, the decision between a biological and mechanical prosthesis is based on medical assessment and patient preference. The following are important factors in this decision: biological and chronological age, life expectancy, and absolute or relative contraindications to the use of oral anticoagulants after surgery, e.g., comorbidities or intense sport activity. The 2014 American Guidelines^22^ recommend a mechanical prosthesis for mitral or aortic valve implantation in patients less than 60 years old who have no contraindication to the use of oral anticoagulants (recommendation IIa, evidence level B); a bioprosthesis is recommended for those aged over 70 years, and biological or mechanical prostheses are recommended for patients between 60 and 70 years of age (both with recommendation IIa and evidence level B).^22^ The 2012 European directive recommends the use of a mechanical prosthesis in patients less than 60 years old in the aortic position and in those under 65 in the mitral position (recommendation IIa, evidence level C).^23^ Therefore, there is currently no exact recommendation for the choice of prosthesis in the 60-70 year age range, and there is no solid evidence upon which the choice of one prosthesis over another can be made. Thus, variability in preferences will likely occur among patients, in special for those aged between 60 and 70 years, and the data from this systematic review should be useful to inform the decision.^24^

Randomized studies to assess treatments for valvular heart disease pose unique clinical challenges in cardiology for several reasons. First, the disease is of relatively low prevalence. Second, comparing surgical complex interventions in randomized controlled trials is difficult. Third, important clinical endpoints are assessed only after decades of follow-up. Fourth, continuing advances in prosthetic heart valve technology make follow-up a moving target because long-term data by definition are available only for older prostheses. Newer tissue and mechanical prostheses afford superior hemodynamics compared with their older counterparts, and data suggest that durability and patient mortality are superior with newer compared with older bioprostheses. In parallel, the mechanical prosthesis has also evolved. Nevertheless, important advances have been made through the results of randomized trials in equally challenging fields in cardiology, for instance, assessment of CABG vs medical treatment or percutaneous treatment. It is in the public interest, both in health and financial terms, to have access to high-quality data to inform decisions regarding the use of health technologies. Therefore, more and better trials comparing technologies for patients with valvular heart disease are needed and feasible. Funding for those trials might be provided by the prosthesis industry had the regulatory environment enforced formal comparative testing, as is currently done with drugs. Alternatively, public funding agencies might support these trials.

### Evidence Applicability

Bleeding was more common in the mechanical prosthesis group than in the biological prosthesis group. However, the studies included in the present review were conducted at a time predating the International Normalised Ratio (INR) and the International Sensitivity Index (ISI). The INR was introduced in the 1980s, and the ISI was introduced in the 1990s. It is possible that with the improved anticoagulation monitoring processes currently available, the difference in the risk of bleeding for patients treated with mechanical versus biological prostheses may be lower than that found in this review.

In our systematic review, three of the four trials considered used the first generation biological prostheses and single disc mechanical prostheses.^13^^,^^17^^,^^20^ Although uncontrolled studies suggest that second and third generation biological prostheses have greater durability,^24^ in the study by Stassano et al.,^21^ which included both modern biological and modern mechanical prostheses, the increased risk of reoperation was similar to that observed in the other trials.

### Concordance and discordance in relation to other studies

We found a single meta-analysis that included three trials comparing old generation biological and mechanical prostheses, which was published by Kassai et al.^13^ 15 years ago. In the current review, we identified an additional study^21^ that compared modern prostheses. In addition, the randomized studies of the Veterans Affairs group^14^ and the Edinburgh group^18^ presented new publications with extended follow-up periods of 15 and 20 years, respectively.^17^^,^^20^ Our results, as well as adding an additional study, reflect long-term follow-up, which is fundamental for better characterising the clinical progress of patients undergoing prosthetic valve implantation.

Indeed a number of observational studies have shown the extended durability of biological prostheses, with a decrease in mortality of reoperation.^5^ In parallel, use of biological prostheses has increased substantially.^6^ However, the evidence provided by observational studies is weak due to the high risk of selection bias. Conversely, observational studies have also suggested increased mortality with biological prosthesis for mitral valve replacement. Our results showed a nonsignificant trend towards increased mortality with biological valves irrespective of position.

### Quality of evidence (GRADE)

The included randomized studies present a low risk of bias and directly evaluate whether differences in clinical outcomes exist between biological and mechanical prostheses. Reporting bias is also unlikely. Regarding the mortality, reoperation and bleeding outcomes, the estimated effect of biological versus mechanical prostheses exhibited good precision and absence of serious inconsistency. We, therefore, consider that the evidence is of high quality (Table 3). For the systemic arterial embolism and endocarditis outcomes, although there was no serious inconsistency, the estimated effect is imprecise (i.e., the 95% CI is compatible with an unfavourable outcome of both the bioprosthesis and the mechanical prosthesis).

**Table 3:** Assessment of the quality of evidence and summary of findings

Quality assessment						Summary of findings	
No of studies (No. of participants)	Study limitations	Inconsistency	Indirectness	Imprecision	Publication bias	Relative risk (95% CI)	Quality
**Mortality**							
4 (1,535)	No serious limitations	No serious inconsistency	Direct	No serious imprecision	Unlikely	1.07 (0.99, 1.15)	⊕⊕⊕⊕ HIGH
**Reoperation**							
4 (1,535)	No serious limitations	No serious inconsistency	Direct	No serious imprecision	Unlikely	3.60 (2.44, 5.32)	⊕⊕⊕⊕ HIGH
**Bleeding**							
4 (1,535)	No serious limitations	No serious inconsistency	Direct	No serious imprecision	Unlikely	0.64 (0.52, 0.78)	⊕⊕⊕⊕ HIGH
**Embolism**							
4 (1,535	No serious limitations	No serious inconsistency	Direct	Imprecision^†^	Unlikely	0.93 (0.66, 1.31)	⊕⊕⊕O MODERATE
**Endocarditis**							
4 (1,535)	No serious limitations	No serious inconsistency	Direct	Imprecision*	Unlikely	1.21 (0.78, 1.88)	⊕⊕⊕O MODERATE

* Effect estimate compatible with either no effect or harm; † Effect estimate compatible with either substantial benefit or harm.

† Effect estimate compatible with either substantial benefit or harm.

### Strengths and weaknesses

Our systematic review has strengths and limitations. The development of the search strategy may be cited as a strength, as it was very sensitive and offered little likelihood of not identifying any relevant evidence. The main databases were searched along with unpublished evidence sources, and a manual evidence search was performed. All systematic review procedures were directed by guidelines and literature specific to this type of study, including all methodological characteristics necessary for proper execution of the review.^10^ The included trials conducted extended follow-up of patients (from 2 to 20 years), allowing adequate evaluation of the effect of biological versus mechanical prostheses in clinical outcomes, particularly those with late incidence of outcomes such as the need for reoperation.

With regard to limiting factors, the inherent limitations of systematic reviews should be considered, such as slight differences in the populations of trial studies. For example, patients with a small aortic annulus were excluded in the Bloomfield study,^20^ those with a small mitral annulus or significant coronary artery disease were excluded from the Veterans study,^17^ and patients with aortic valve lesions were excluded from Vallejo's study.^13^

A major weakness of our systematic review is the age of available trials. Three of the 4 trials included are old and used first generation biological prostheses and single-disk mechanical prostheses. As both prostheses and ancillary care have evolved, it is possible that the results we have observed would not be currently applicable. Indeed a number of observational studies have shown the higher durability of biological prostheses and a trend towards its use in younger patients.^6^ However, the evidence provided by observational studies is weak due to the high risk of selection bias. Furthermore, the results of the randomized trial by Stassano et al.^21^ comparing modern biological to mechanical prosthesis are completely consistent with those of previous trials. In special, there was an important increase in the need of reoperation and a decreased risk of bleeding with biological prostheses. Thus, although more evidence from new trials comparing biological to mechanical is urgently needed, the best available evidence does not support the increasing preference for biological prostheses.

## Conclusion

Our systematic review of randomized studies, which evaluated the outcomes of patients who randomly received biological and mechanical valve prostheses, showed that although there are no differences in mortality, there is a significant increase in the risk of new valve replacement surgery when opting for biological prostheses.

In contrast, the risk of bleeding is lower with bioprostheses. There were no differences in mortality, the risk of endocarditis or systemic embolism between the two prosthesis types. Although three of the four trials included in our meta-analysis used old generation biological and mechanical prostheses, the trial which evaluated currently used prostheses for aortic valve replacement showed the same results. Nevertheless, evidence to inform the choice between currently available prostheses is very limited and mostly based on observational studies. Randomized comparisons are utterly necessary.
